# Adherence to the iDSI reference case among published cost-per-DALY averted studies

**DOI:** 10.1371/journal.pone.0205633

**Published:** 2019-05-01

**Authors:** Joanna Emerson, Ari Panzer, Joshua T. Cohen, Kalipso Chalkidou, Yot Teerawattananon, Mark Sculpher, Thomas Wilkinson, Damian Walker, Peter J. Neumann, David D. Kim

**Affiliations:** 1 Center for the Evaluation of Value and Risk in Health, Tufts Medical Center, Boston, MA, United States of America; 2 Institute of Global Health Innovation, Imperial College London, London, United Kingdom; 3 The Saw Swee Hock School of Public Health, National University of Singapore, Singapore; 4 Centre for Health Economics, University of York, York, United Kingdom; 5 Health Economics Unit, School of Public Health and Family Medicine, University of Cape Town, South Africa; 6 Bill & Melinda Gates Foundation, Seattle, WA, United States of America; University of Exeter, UNITED KINGDOM

## Abstract

**Background:**

The iDSI reference case, originally published in 2014, aims to improve the quality and comparability of cost-effectiveness analyses (CEA). This study assesses whether the development of the guideline is associated with an improvement in methodological and reporting practices for CEAs using disability-adjusted life-years (DALYs).

**Methods:**

We analyzed the Tufts Medical Center Global Health CEA Registry to identify cost-per-DALY averted studies published from 2011 to 2017. Among each of 11 principles in the iDSI reference case, we translated all methodological specifications and reporting standards into a series of binary questions (satisfied or not satisfied) and awarded articles one point for each item satisfied. We then calculated methodological and reporting adherence scores separately as a percentage of total possible points, measured as normalized adherence score (0% = no adherence; 100% = full adherence). Using the year 2014 as the dissemination period, we conducted a pre-post analysis. We also conducted sensitivity analyses using: 1) optional criteria in scoring, 2) alternate dissemination period (2014–2015), and 3) alternative comparator classification.

**Results:**

Articles averaged 60% adherence to methodological specifications and 74% adherence to reporting standards. While methodological adherence scores did not significantly improve (59% pre-2014 vs. 60% post-2014, p = 0.53), reporting adherence scores increased slightly over time (72% pre-2014 vs. 75% post-2014, p<0.01). Overall, reporting adherence scores exceeded methodological adherence scores (74% vs. 60%, p<0.001). Articles seldom addressed budget impact (9% reporting, 10% methodological) or equity (7% reporting, 7% methodological).

**Conclusions:**

The iDSI reference case has substantial potential to serve as a useful resource for researchers and policy-makers in global health settings, but greater effort to promote adherence and awareness is needed to achieve its potential.

## Background

Policy makers and program managers, particularly those in low- and middle-income countries (LMIC), often face prioritization decisions with limited resources [1]. Economic evaluation, such as cost-effectiveness analyses (CEA), can provide insight into the comparative value of various health interventions and therefore help inform priority setting [2].

Since the original Panel on Cost‐Effectiveness in Health and Medicine proposed the use of a reference case as a benchmark of quality and methodological rigor [3, 4], various guidelines for conducting economic evaluation have been proposed [5, 6]. The Consolidated Health Economic Evaluation Reporting Standards (CHEERS) Checklist, a widely cited reporting guideline, is used to ensure study results are reported with clarity and accuracy, yet does not provide methodological guidelines for how analyses should be conducted [7]. Many countries, particularly high-income ones, have also developed their own reference cases to inform decision-making in their health care systems [8–11].

In contrast, most low- and middle-income countries (LMICs) have not developed such guidelines, possibly due to their limited capacity to do so [12]. In fact, only 12 LMICs currently have economic evaluation guidelines specific to their country [13]. Although the general principles of guidelines for high-income countries can still be applied to LMICs, variations in both approaches and methods used limit their usefulness. For example, most high-income country guidelines suggest health outcomes be measured using quality-adjusted life-years (QALYs). The estimation of QALYs requires a preference weight for different health states, called health-related quality of life, on which LMICs often have limited data.

To address the need for a reference case that could broadly apply to different contexts, particularly in LMICs, the Bill and Melinda Gates Foundation (BMGF) supported the development of the Gates Reference Case to ensure high quality and transparent CEA in global health priority setting [14]. One of the key recommendations is to support the use of disability-adjusted life years (DALY), as disability weights are more readily available and more easily transferable across different countries [15]. The first version was published in 2014 as the Gates Reference Case and, later in 2016, was renamed the International Decision Support Initiative (iDSI) Reference Case to convey the breadth of its intended applicability [14, 16].

The iDSI reference case fills a major gap in global health economics, as it is the only resource of economic evaluation best practices for many policymakers in LMICs looking for guidance on resource prioritization. However, no study has assessed whether the development of the guideline is associated with an improvement in research practice for CEAs employing DALYs. This paper aims to quantify the methodological and reporting quality of cost-per-DALY averted studies over time, as measured by adherence to best practices enumerated by the iDSI reference case.

## Methods

### Data

#### The iDSI reference case

The iDSI reference case includes 11 principles: transparency, comparator, evidence, measures of health outcome, costs, time horizon/discount rate, perspective, heterogeneity, uncertainty, budget impact, and equity considerations. Each principle has a number of corresponding methodological specifications and reporting standards. By using this tiered structure, the reference case aims to serve as a framework that provides best practice guidance while allowing for flexibility depending on context [16], and thus is the most appropriate economic evaluation guideline for LMICs without their own national guidelines.

#### Global health CEA registry

We analyzed data from the Tufts Medical Center Global Health CEA Registry, a continually updated database of English-language economic evaluations in the form of cost-per-DALYs averted [17]. Among 620 cost-per-DALY averted studies in the database, we selected all articles published three years before and after the initial release of the iDSI reference case (2011–2017) to examine the impact of its publication on the literature (N = 398). We focused particularly on economic evaluations using the DALY metric because it is recommended as a main outcome metric by the iDSI reference case and it is used more often as a health outcome measure in LMICs than equivalent metrics such as the QALY [16, 18].

To ensure a comprehensive assessment of adherence to the reference case, two independent readers (JE and AP) extracted additional information from each study in our sample using REDCap, an online data collection platform [19], including data on: currency reported; subgroup analyses conducted; limitations reported; structural sensitivity analyses conducted; budget impact conducted; justification of alternative methodology; and comparator setting.

### Adherence score

We first translated all 30 methodological specifications and 38 reporting standards (across 11 principles) listed in the reference case into questions with discrete binary outcomes (standard satisfied or standard not satisfied) (Table 1). We then designated reference case elements as “required” or “optional” based on our interpretation of the language in the report (Table A in S1 File). We deemed 19 methodological specifications and 21 reporting standards “required”.

**Table 1:** Evaluation criteria for adherence to the iDSI reference case.

Reference case principle	Methodological specification evaluation question		Reporting standard evaluation question	
Transparency	Decision problem, limitations, and declarations of interest are appropriately characterized.	Decision problem characterized?	Decision problem (population, intervention, comparator, outcome), evaluation's limitations, and declarations of interest are fully described.	Population stated?
		Limitations characterized?		Intervention stated?
		Declaration of interest reported?		Comparator stated?
				Outcome stated?
				Limitations stated (general)?
				Conflict of interest statement included?
				Funding source stated?
Comparator(s)	Intervention(s) currently offered to the population (standard of care) is the base case comparator.	Comparator is standard of care?	Comparator and its availability are clearly stated, and outcomes reported in incremental cost effectiveness ratio.	Comparator clearly stated?
				Reported ICER?
Evidence	Systematic literature review is used as source of evidence.	Systematic review used?	Methods of evidence collection are stated and sources of parameters are cited.	Parameter sources stated?
				Parameter sources cited?
Measure of health outcome	DALYs are used as the base case outcome measure.	DALYs as main outcome?	Methods for weighting of DALYs are stated.	Weighting methods stated?
Costs	Costs are relevant to the context and stated perspective, and include implementation costs.	Costs are true to reported perspective?	Costs are reported in local currency and USD.	Costs in local currency?
		Costs include implementation?		Costs in USD?
Time horizon and discount rate	Lifetime time horizon and 3% discount rate for costs and outcomes are used in base case.	Lifetime time horizon used?	Time horizon and discount rate are clearly stated.	Time horizon clearly stated?
		3% discount rate used?		Discounting for both costs and outcomes clearly stated?
		Discount rate used for costs and effects?		
Perspective	Societal perspective is used in base case, and relevant costs to this perspective (including direct health costs) are included.	Limited societal perspective used?	Perspective and base case outcomes are clearly stated.	Perspective clearly stated?
		Direct health costs reported?		
Heterogeneity	Heterogeneity is analyzed for appropriate subgroups.	Subgroup analysis performed/stated?	Subgroup characteristics and analysis of heterogeneity are clearly described.	Subgroup analysis performed/stated?
Uncertainty	Sensitivity analyses are performed on parameter source uncertainty (deterministic), parameter precision (probabilistic), and analysis structure (structural).	Structural sensitivity analysis performed?	Magnitude of uncertainty in the model's structure, parameters, and precision are reported.	Reported results of sensitivity analysis?
		Sensitivity analysis of parameter source performed (deterministic)?		
		Sensitivity analysis of parameter precision performed (probabilistic)?		
Budget impact	Intervention(s) budget impact is assessed.	Budget impact assessment performed?	Intervention(s) budget impact is reported.	Impact on budget stated?
Equity considerations	Intervention(s) implications on equity are assessed.	Equity addressed at all in the paper?	Intervention(s) implications on equity are stated.	Influence of equity considerations stated in the paper?

DALY: disability-adjusted life year; QALY: Quality-adjusted life year; LY: life year; USD: United States dollar; ICER: incremental cost-effectiveness ratio.

Evaluation questions scored as either 0 (item not satisfied) or 1 (item satisfied), and are each weighted equally. Optional requirements noted in Table A in S1 File and are only included in sensitivity analysis scoring.

Our base-case analysis examined adherence scores consisted only of “required” elements. We evaluated each published cost-per-DALY averted study’s adherence to methodological specifications (0–19 items) and reporting standards (0–21 items). We then separately calculated reporting and methods raw scores as a percentage of total possible points, measured as normalized adherence score (0% = no adherence, i.e., no requirements adhered to; 100% = full adherence, all requirements adhered to).

### Analysis

#### Descriptive analysis

We examined the association between adherence score and certain study characteristics, including whether the study cited the reference case, the study funder characteristics, and journal attributes. We categorized study funders into the following groups (not mutually exclusive): academic, government, healthcare organization, industry, intergovernmental organization, BMGF, non-BMGF, and other. We also stratified selected articles into clinical versus non-clinical journals using SCImago Journal Rank’s subject categorization (medicine vs. health policy, public health, non-health) [20]. Finally, we recorded 2016 journal impact factor quartiles and categorized studies as high impact (first quartile), medium impact (second quartile), or low-impact (third and fourth quartiles) [20].

#### Statistical analysis

To examine whether the iDSI guideline has, since its release in 2014, improved the methodological and reporting practices of cost-per-DALY averted studies, we calculated mean adherence scores by year from 2011 to 2017. We conducted a pre-post analysis of improvement in methodological and reporting adherence scores. As the reference case was first released in January of 2014 [21], we considered that year to be the reference case’s dissemination period, and hence did not include articles published during that year in our pre-post analysis. We also compared the overall methodological and reporting adherence scores, stratified by the 11 principles.

#### Sensitivity analysis

We conducted three sensitivity analyses. First, we included the “optional” criteria in the calculation of adherence scores for a random 10% subset of the articles to explore the impact of including optional items in the adherence score. Second, given that efforts to increase awareness of new guidelines may take longer than one year, and subsequent development and publication of adherent CEAs can span more time, we conducted a sensitivity analysis to explore alternate dissemination period lengths. Primarily, we expanded the dissemination period from 2014 to 2014–2015 to examine the influence of a longer dissemination period on adherence. Third, we used an alternative classification to determine adherence to the comparator principle’s corresponding methodological specification. In our base case analysis, we designated an article adherent to the iDSI’s comparator methodological specification only if the article explicitly reported their comparator as the “standard of care”. In this sensitivity analysis, we classified an article as adherent so long as it specified a comparator other than “do nothing” or some other non-action. To be consistent with the iDSI reference case principle that the standard of care must include at least “minimal supportive care” [22], we designated “do-nothing” interventions as non-adherent.

## Results

### Descriptive statistics

Among 398 cost-per-DALY averted studies published from 2011–2017, 215 (54%) focused on LMICs and 263 (68%) targeted communicable diseases, such as diarrhea, HIV/AIDs, tuberculosis, and malaria (Table 2). Articles averaged 60% adherence to the reference case’s methodological specifications and 74% adherence to reporting standards. Table 3 summarized iDSI Reference case normalized adherence scores by year, sponsor, and journal aspects (The raw scores are available from Table B in S1 File). No article achieved full adherence to either the methodological specifications or the reporting standards.

**Table 2:** Characteristics of cost-per-DALY averted studies published 2011–2017.

GBD Super Region	Number of studies	% of the sample
Sub-Saharan Africa	125	31.4
High Income	66	17.0
Multiple Regions #	52	13.1
Southeast Asia, East Asia, and Oceania	45	11.6
South Asia	36	9.3
Latin America and Caribbean	33	8.5
N/A	22	5.7
North Africa and Middle East	10	2.6
Central Europe, Eastern Europe, and Central Asia	9	2.3
Intervention*		
Pharmaceutical	112	28.1
Immunization	106	26.6
Care delivery	74	18.6
Health education or behavior	73	18.3
Screening	63	15.8
Surgery	36	9.1
Other	34	8.5
Medical procedure	15	3.8
GBD Disease Category		
Other	90	22.6
Diarrhea, LRI, and other common infectious diseases	79	21.1
HIV/AIDS and tuberculosis	79	21.1
Neglected tropical diseases and malaria	41	11.0
Mental and behavioral disorders	28	7.5
Other communicable, maternal, neonatal, and nutritional disorders	25	6.7
Cardiovascular and circulatory disease	24	6.4
Diabetes, urogenital, blood, and endocrine disorders	16	4.3
Neoplasms	12	3.2
Digestive diseases	4	1.1
Study sponsor*		
Government	153	38.4
Foundation	124	31.2
Academics	53	13.3
Intergovernmental Org	41	10.3
Other	24	6.0
Healthcare Org^	23	5.8
Industry	16	4.0

# “Multiple regions”: studies that reported cost-effectiveness estimates for countries in different regions.

* Not mutually exclusive. GBD: Global burden of disease.

^ Health care organizations include insurance companies, hospitals. LRI: Lower respiratory infection.

Source: Tufts Medical Center Global Health Cost-Effectiveness Registry (www.ghcearegistry.org)

**Table 3:** iDSI reference case adherence scores by year, sponsor, and journal aspects.

		Methodological adherence: Normalized score (out of 100)			Reporting adherence: Normalized score (out of 100)		
	N	Mean (SD)	Min	Max	Mean (SD)	Min	Max
**Base case analysis**	398	59.6 (11.5)	26.3	89.5	73.9 (8.5)	42.9	90.5
Pre-post period^1^							
Pre-period: 2011–2013	138	58.9 (12)	26.3	89.5	72.3 (9.1)*	42.9	90.5
Post-period: 2015–2017	213	59.7 (11.4)	26.3	89.5	74.9 (7.9)*	47.6	90.5
Study sponsor^2^							
Academic	53	59.7 (12.7)	36.8	84.2	75 (8.2)	52.4	90.5
Government	153	60.7 (11)	31.6	89.5	75.4 (7.5)	47.6	90.5
Healthcare Org	23	65.4 (11)	31.6	89.5	77.2 (8.1)	57.1	90.5
Industry	16	60.5 (11.2)	31.6	73.7	74.7 (7.3)	57.1	90.5
Intergovernmental	41	61.9 (10.3)	36.8	84.2	74.4 (7.6)	61.9	90.5
Foundation	56	60.2 (12)	36.8	89.5	74.1 (8.2)	57.1	90.5
BMGF	74	60.1 (11)	31.6	84.2	75.4 (8.5)	47.6	90.5
Other	24	58.6 (13.5)	31.6	84.2	73.2 (8.9)	52.4	90.5
Cite reference case							
Yes	9	62.0 (12.6)	47.4	89.5	78.8 (7.6)	71.4	90.5
No	251	59.9 (11.2)	26.3	84.2	74.5 (8.0)	47.6	90.5
Journal type							
Clinical	318	60.3 (11)*	26.3	89.5	74.2 (7.9)	47.6	90.5
Non-clinical	80	56.8 (13.1)*	26.3	78.9	72.5 (10.2)	42.9	90.5
Journal impact factor^3^							
High	336	60.5 (11.2)*	26.3	89.5	74.1 (8.6)	42.9	90.5
Medium	45	56.1 (12.7)	26.3	73.7	73 (7.5)	57.1	90.5
Low	12	50 (6.9)*	42.1	63.2	70.6 (9.7)	52.4	85.7
**Sensitivity analyses**							
#1: Inclusion of "optional” elements							
Base case analysis	398	59.6 (11.5)	26.3	89.5	73.9 (8.5)	42.9	90.5
10% random sample^4^	40	46.2 (8.3)	23.3	66.7	52.4 (5.6)	39.4	63.2
#2: Alternate dissemination period							
Pre-period: 2011–2013	138	58.9 (12)	26.3	89.5	72.3 (9.1)	42.9	90.5
Post-period: 2016–2017	135	59.2 (11.6)	26.3	84.2	74.6 (7.4)	57.1	90.5
#3: Alternate comparator classification^5^							
Base case: standard of care	398	59.6 (11.5)	26.3	89.5	N/A		
Use of any comparator	398	60.4 (11.7)	26.3	89.5	N/A		

*: Statistically significant difference (p<0.05) between categories (within methods/reporting requirements) per Student's t-test

1: Year 2014 was excluded from pre-post analysis to serve as dissemination period.

2: Categories are not mutually exclusive, t-test not calculated.

3: Journal impact factor categories defined by 2016 SCImago Journal Rank quartile: high = first quartile; medium = second quartile; low = third and fourth quartiles. Five journals' impact factors were not available.

4: Base case scoring only included reference case elements designated as “required” per report language. “Optional” elements reintroduced in sensitivity analysis of random subsample of 10% of articles.

5: Any listed comparator scored as adherent, unless comparator was “do-nothing”.

Of the 213 articles published after 2014 (i.e. 2015–2017), only 9 (4%) cited the iDSI reference case. For articles that did so, adherence to reporting standards averaged 79%, five percentage points higher than mean adherence for the full sample, while adherence to methodological specifications did not differ from adherence for the full sample. Funding source (BMGF vs. non-BMGF) was not significantly associated with a change in adherence scores for either methodological (mean score of 60% vs. 60%) or reporting (mean score of 75% vs. 74%).

Studies published in clinical journals had marginally higher adherence (60% methodological adherence, 74% reporting adherence) than studies in non-clinical journals (57% methodological adherence, 73% reporting adherence). On average, methodological adherence scores for articles published in high-impact journals exceeded the corresponding scores for studies published in low-impact journals (61% vs. 50%); for reporting adherence, the corresponding difference was 74% vs. 71%.

**Fig 1:**
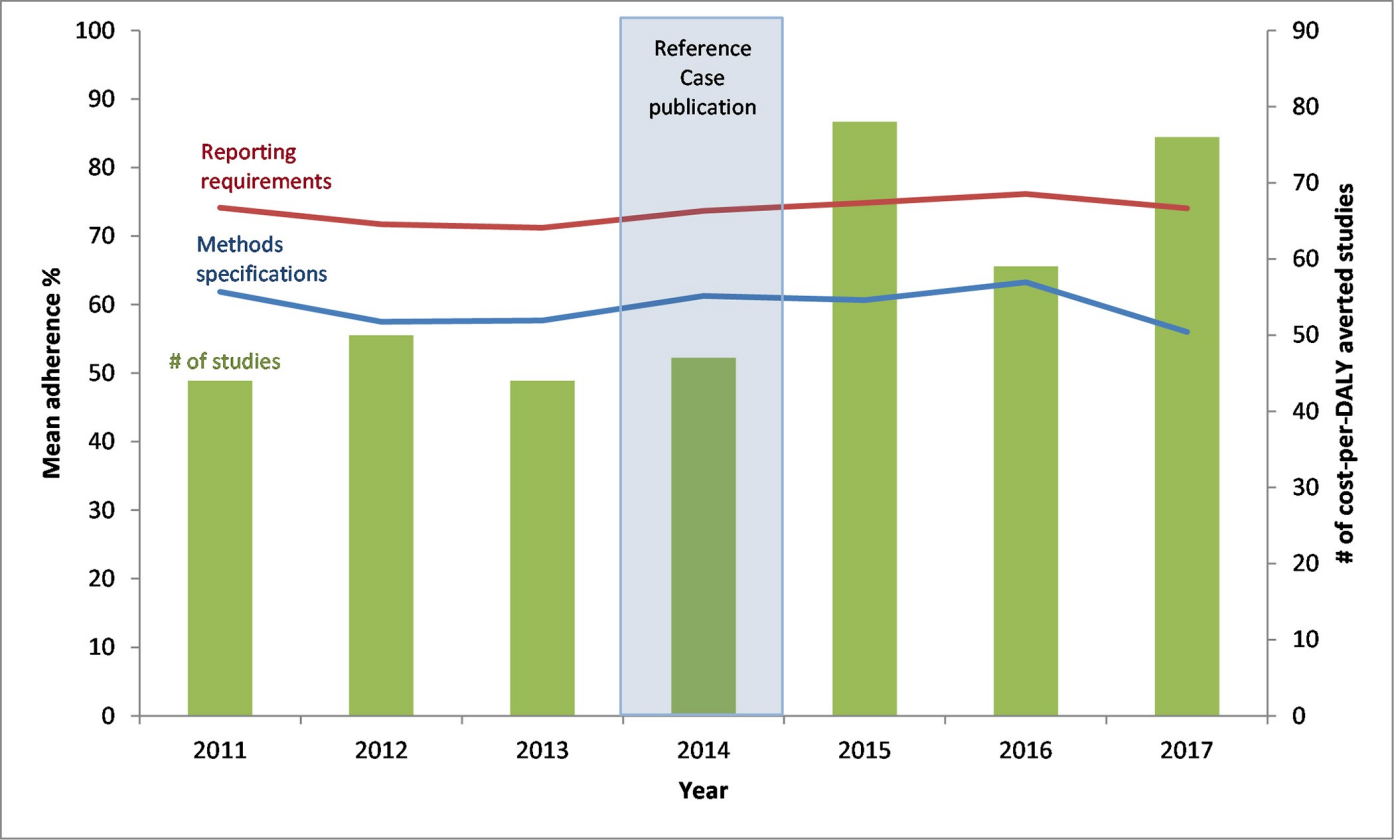
iDSI reference case adherence scores and number of cost-per-DALY averted studies over time.

Methodological adherence did not improve after publication of the reference case compared to the pre-2014 period (59% adherence pre-2014 vs. 60% post-2014, p = 0.53). Reporting standard adherence slightly increased (72% adherence pre-2014 vs. 75% post-2014, p<0.01) (Fig 1 and Table C in S1 File).

### Methodological adherence versus reporting adherence scores

**Fig 2:**
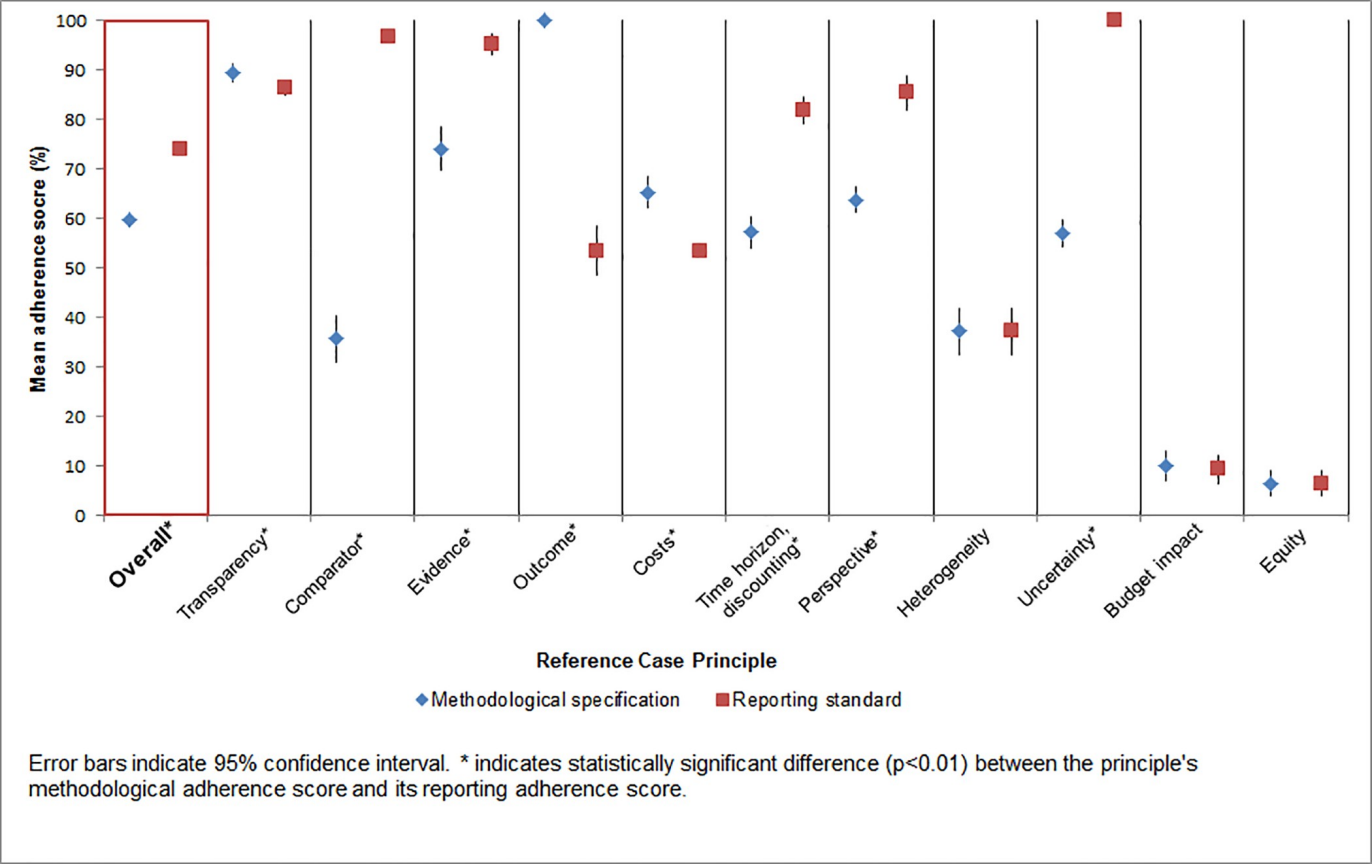
Methodological vs. reporting adherence scores: Overall and by each principle.

Across the 11 principles, reporting standard adherence exceeded methodological specification adherence by 14 percentage points (74% vs. 60%). Reporting adherence score were highest for the following principles: uncertainty (mean of 100%), comparator (97%), and evidence (95%). Methodological adherence scores were highest for the outcome measure (100%), transparency (89%), and evidence (74%) principles (Fig 2).

Methodological adherence scores were higher than reporting adherence scores for the following principles: transparency (89% methodological adherence score vs. 86% reporting adherence score), outcome (100% vs. 54%), and costs (65% vs. 54%). Reporting adherence scores exceeded methodological adherence scores for the following principles: comparator (36% methodological adherence score vs. 97% reporting adherence score), evidence (74% vs. 95%), time horizon/discounting (57% vs. 82%), perspective (64% vs. 85%), and uncertainty (57% vs. 100%) (Fig 2). Articles seldom addressed the principles of budget impact (10% methodological adherence score, 9% reporting) or equity (7%, 7%) (Fig 2).

### Sensitivity analyses

Inclusion of optional criteria in our adherence score calculation decreased mean methodological adherence by 14 percentage points (60% to 46%) and mean reporting adherence by 22 percentage points (from 74% to 52%). When we increased the dissemination period to 2014–2015 (base case: 2014), we found no change in our results. Using an alternate comparator principle classification (base case: comparator must be standard of care; alternative: comparator can be any intervention other than “do-nothing”) also had little impact.

## Discussion

Since its introduction in 2014, adherence to the iDSI reference case among published cost-per-DALY averted studies has improved for reporting standards, but not for methodological specifications. Adherence to the reference case’s reporting standards exceeds adherence to its methodological specifications, perhaps reflecting the relative ease of revising the way information is presented and greater effort needed to conform to analytic requirements. Moreover, other reporting guidelines, such as CHEERS [7] or country-specific recommendations, may have independently promoted more rigorous reporting, with the unintended effect of boosting adherence to the iDSI reference case.

However, methodological and reporting adherence scores varied substantially across reference case principles, demonstrating ways in which articles are falling short of guidelines. For example, articles almost always report their comparator clearly (as recommended by reporting standards), but do not necessarily specify whether the comparator is considered standard of care (as recommended by methodological specifications). Similarly, all articles reported findings from sensitivity analyses, but did not always conduct comprehensive structural, probabilistic, and deterministic sensitivity analyses.

In some cases, methodological adherence exceeded reporting adherence. For example, articles often included implementation costs (as recommended by methodological specifications), but did not as frequently report these costs in both US dollars and local currency (as recommended by reporting standards). Because the methodological specifications and reporting standards address distinct issues, future guidelines should continue to include recommendations for both types.

It is important to consider what level of adherence should be seen as satisfactory. Although articles in our sample were more adherent to reporting guidelines, they adhered to just over half of methodological specifications. Adherence scores were notably lower for particular principles—heterogeneity, budget impact, and equity—indicating overall neglect of these issues in cost-per-DALY averted studies. The adherence scores are perhaps best thought of as a baseline against which to measure improvement, and as a call to action to promote higher quality and comparability.

The lack of adherence to the iDSI reference case might reflect the competing influences of other guidelines, as authors may prioritize adherence to local guidelines that are more relevant to their context [3, 11]. For example, the South African pharmacoeconomic guidelines recommend a base case 5% discount rate, which differs from the 3% value recommended by the reference case [23]. Although the iDSI reference case supports the use of alternative discount rates where appropriate to the decision problem and constituency, published CEAs that adhere to the local guidelines may be scored as non-adherent to the methodological specifications in this analysis.

Another possible explanation for relatively low adherence for certain items is that authors may not be aware of the guidelines. We found that only 4% of the identified studies published after 2014 directly cited the iDSI reference case. The BMGF and iDSI have focused educational campaigns on national payers and health technology assessment (HTA) agencies in LMICs, rather than on researchers, who are primary authors of published studies [22]. Future studies should examine whether the reference case has influenced country-specific guidelines, such as Thailand’s HTA assessment guideline [9].

### Limitations

The primary limitation of our study is that the post-evaluation period (2015–2017) may not have been sufficiently long to detect the impact of the reference case. Though it was initially released in early 2014, as noted, the iDSI reference case was not officially published in an academic journal until 2016 [16]. However, dissemination efforts began in 2013 at a BMGF-hosted workshop for multi-sectoral stakeholders, which was later considered “a major part of the Gates reference case development”[14]. Although more time may be needed for the field to adopt these guidelines, as new CEAs can take years to conduct and publish, we believe our results on adherence to the iDSI reference case can serve as a baseline estimate. Adherence should be re-analyzed in the future as the field continues to grow.

Furthermore, our use of dichotomous (i.e., “yes/no”) questions to score adherence may be inconsistent with the more nuanced goals of the iDSI reference case. Because the reference case is designed to be applicable in a range of different country-specific contexts, it must balance the goals of study comparability and quality against the goal of local applicability [6, 22, 24]. To address this limitation, we omitted “optional” standards from our adherence calculation for the base case. That is, we assumed that the “optional” elements represent conditional requirements intended by the reference case authors to allow for local adaptability. Our sensitivity analysis that included all elements in our calculation of the adherence score (i.e. both the “required” and “optional” elements) yielded lower adherence scores.

Assessing adherence to the comparator principle posed a particular challenge because this assessment requires subjective judgment on whether the specified comparator constitutes the “standard of care.” Although our sensitivity analysis of altering the definition of appropriate comparator had little impact on our findings, a “do nothing” intervention, which is deemed inappropriate by the iDSI’s comparator methodological specification, can be regarded as “standard of care” for some conditions in some settings, such as a population screening program for tuberculosis [25, 26].

Also, our findings cannot be generalized to the rest of the economic evaluation literature as the Tufts Medical Center Global Health CEA Registry catalogs only published cost-per-DALY averted studies. For example, our analysis excluded gray literature (i.e., material not disseminated in regularly published, indexed journals). Gray literature may be more prevalent in some countries, especially those without local guidelines.

Finally, our approach for scoring articles inherently involves reviewer judgment to determine author intent and to resolve ambiguities (e.g., determining whether the comparator is “clearly” stated). We attempted to mitigate this problem by having two reviewers read each article and, in cases of no consensus, we appealed to a third reviewer.

### Policy implications

As posited by Nugent and Briggs, future research on the subject should ask, “what specific help does the iDSI reference case offer the analyst, who, while attempting to conform to the principles, nevertheless has to choose and implement the methods?” [27] It is possible that the methodological guidelines impose an excessive burden on researchers, raising “issues about the resources and data requirements to meet the principles” [22].

Future qualitative research can focus on researcher consideration of best practice guidelines in study design and reporting, and on how to increase guideline acceptance among authors. Studies could also further evaluate the methods and reporting adherence for articles that strongly adhere to the iDSI reference case, as these analyses may serve as useful examples for other CEA authors. Analysis of the impact of the reference case on perceived quality and usefulness of economic evaluations by decision makers would be useful.

Moving from guideline development to implementation is a vital step towards improving the quality of economic evidence in global health. Future efforts could include additional educational workshops for researchers, students, and policymakers. Policymakers and major funders of economic evaluations, such as the BMGF, could require that researchers adhere to reference case recommendations in grant applications. Journals and reviewers should also impose high-quality standards for economic evaluations. Moving forward, journals may require reviewers to fill out a rubric similar to the instrument in our study that measures the adherence of economic evaluations to the iDSI reference case guidelines.

## Conclusion

Since its initial launch in 2014, our study indicates that the development of the iDSI reference case is associated with improving reporting standards for economic evaluation focused on global health, but no improvement in methodological practice. Although the reference case has substantial potential to serve as a resource for researchers and policy makers in global health and economics, more effort to promote adherence and awareness may be needed.

## Supporting information

S1 File Supplementary materials. **Table A:** Full instrument for evaluating adherence to the iDSI reference case;
**Table B:** iDSI Reference Case adherence raw scores by year, sponsor, and journal aspects;
**Table C:** iDSI Reference case adherence scores by year.
(DOCX)

S2 File Study datasets. (DTA)

## References

[1] Glassman A, Chalkidou K, Giedion U, Teerawattananon Y, Tunis S, Bump JB, et al Priority-setting institutions in health: recommendations from a center for global development working group. Glob Heart. 2012;7(1):13–34. Epub 2012/03/01. 10.1016/j.gheart.2012.01.007 . 25691165

[2] Chalkidou K, Glassman A, Marten R, Vega J, Teerawattananon Y, Tritasavit N, et al Priority-setting for achieving universal health coverage. Bulletin of the World Health Organization. 2016;94(6):462 10.2471/BLT.15.155721 27274598 PMC4890204

[3] Weinstein MC, Siegel JE, Gold MR, Kamlet MS, Russell LB. Recommendations of the Panel on Cost-effectiveness in Health and Medicine. JAMA. 1996;276(15):1253–8. 8849754

[4] Russell LB, Gold MR, Siegel JE, Daniels N, Weinstein MC. The role of cost-effectiveness analysis in health and medicine. JOURNAL-AMERICAN MEDICAL ASSOCIATION. 1996;276:1172–7. 8827972

[5] Sanders GD, Neumann PJ, Basu A, Brock DW, Feeny D, Krahn M, et al Recommendations for conduct, methodological practices, and reporting of cost-effectiveness analyses: second panel on cost-effectiveness in health and medicine. JAMA. 2016;316(10):1093–103. 10.1001/jama.2016.12195 27623463

[6] Murray CJ, Evans DB, Acharya A, Baltussen RM. Development of WHO guidelines on generalized cost‐effectiveness analysis. Health economics. 2000;9(3):235–51. 10790702 10.1002/(sici)1099-1050(200004)9:3<235::aid-hec502>3.0.co;2-o

[7] Husereau D, Drummond M, Petrou S, Carswell C, Moher D, Greenberg D, et al Consolidated health economic evaluation reporting standards (CHEERS)—explanation and elaboration: a report of the ISPOR health economic evaluation publication guidelines good reporting practices task force. Value in Health. 2013;16(2):231–50. 10.1016/j.jval.2013.02.002 23538175

[8] Eldessouki R, Smith MD. Health care system information sharing: a step toward better health globally. Value in Health Regional Issues. 2012;1(1):118–20. 10.1016/j.vhri.2012.03.022 29702818

[9] Teerawattananon Y. Thai health technology assessment guideline development. Journal of the Medical Association of Thailand. 2011;91(6):11. 19253483

[10] Pharmaceutical Benefits Advisory Committee. Guidelines for preparing submissions to the Pharmaceutical Benefits Advisory Committee (PBAC). In: Health Do, editor. Canberra2016.

[11] Rawlins MD, Culyer AJ. National Institute for Clinical Excellence and its value judgments. BMJ: British Medical Journal. 2004;329(7459):224 10.1136/bmj.329.7459.224 15271836 PMC487742

[12] Santatiwongchai B, Chantarastapornchit V, Wilkinson T, Thiboonboon K, Rattanavipapong W, Walker DG, et al Methodological variation in economic evaluations conducted in low-and middle-income countries: information for reference case development. PLoS One. 2015;10(5):e0123853 10.1371/journal.pone.0123853 25950443 PMC4423853

[13] Guide to Economic Analysis and Research (GEAR) Online Resource. Guidelines Comparison: What can I learn from the existing health economic evaluation guidelines? http://www.gear4health.com/gear/health-economic-evaluation-guidelines2019.

[14] Bill WT, Gates M. Foundation Methods for Economic Evaluation Project (MEEP): Final Report NICE International York, UK 2014.

[15] Griffiths UK, Legood R, Pitt C. Comparison of Economic Evaluation Methods Across Low‐income, Middle‐income and High‐income Countries: What are the Differences and Why? Health economics. 2016;25(S1):29–41. 26775571 10.1002/hec.3312 PMC5042040

[16] Wilkinson T, Sculpher MJ, Claxton K, Revill P, Briggs A, Cairns JA, et al The international decision support initiative reference case for economic evaluation: an aid to thought. Value in health. 2016;19(8):921–8. 10.1016/j.jval.2016.04.015 27987641

[17] Center for the Evaluation of Value and Risk in Health. Global Health Cost-Effectiveness Registry. www.ghcearegistry.org/.: Tufts Medical Center,; 2018.

[18] Neumann PJ, Anderson JE, Panzer AD, Pope EF, D'Cruz BN, Kim DD, et al Comparing the cost-per-QALYs gained and cost-per-DALYs averted literatures. Gates Open Research. 2018;2. 10.12688/gatesopenres.12786.1 PMC5801595 29431169

[19] Harris PA, Taylor R, Thielke R, Payne J, Gonzalez N, Conde JG. Research electronic data capture (REDCap)—a metadata-driven methodology and workflow process for providing translational research informatics support. Journal of biomedical informatics. 2009;42(2):377–81. 10.1016/j.jbi.2008.08.010 18929686 PMC2700030

[20] Scimago Journal & Country Rank. Journal Rankings https://www.scimagojr.com/journalrank.php2018

[21] Claxton K, Revill P, Sculpher M, Wilkinson T, Cairns J, Briggs A. The Gates reference case for economic evaluation. Seattle, WA, USA: The Bill and Melinda Gates Foundation 2013.

[22] Gray AM, Wilkinson T. Economic evaluation of healthcare interventions: old and new directions. Oxford Review of Economic Policy. 2016;32(1):102–21.

[23] Matsoso MP. Guidelines for Pharmacoeconomic Submissions. In: Health NDo, editor. 2012

[24] Sculpher M, Pang F, Manca A, Drummond M, Golder S, Urdahl H, et al Generalisability in economic evaluation studies in healthcare: a review and case studies. 2004. 10.3310/hta8490 15544708

[25] Azman AS, Golub JE, Dowdy DW. How much is tuberculosis screening worth? Estimating the value of active case finding for tuberculosis in South Africa, China, and India. BMC medicine. 2014;12(1):216. 25358459 10.1186/s12916-014-0216-0 PMC4224697

[26] Currie CS, Floyd K, Williams BG, Dye C. Cost, affordability and cost-effectiveness of strategies to control tuberculosis in countries with high HIV prevalence. BMC Public Health. 2005;5(1):130. 16343345 10.1186/1471-2458-5-130 PMC1361804

[27] Briggs A, Nugent R. Editorial. Health Economics. 2016;25(Suppl Suppl 1):6–8. 10.1002/hec.3321 PMC5066756. 26804358 PMC5066756

