# Supplementary material for: Adherence to the iDSI reference case among published cost-per-DALY averted studies
Source: PLoS One. 2019 May 1;14(5):e0205633. doi: 10.1371/journal.pone.0205633 (PMC6493721; doi:10.1371/journal.pone.0205633)
Supplement: S1 File — Table A: Full instrument for evaluating adherence to the iDSI reference case; Table B: iDSI Reference Case adherence raw scores by year, sponsor, and journal aspects; Table C: iDSI Reference case adherence scores by year. (DOCX) [file pone.0205633.s001.docx]

**Table A: Instrument for full reference case evaluation**

| **Reference case principle** | **Methodological specification** | | **Reporting standard** | |
| --- | --- | --- | --- | --- |
|  | **Evaluation question** | **Potential max score (base case, sensitivity)** | **Evaluation question** | **Potential max score (base case, sensitivity)** |
| Transparency | Decision problem characterized? | 3, 3 | Population stated? | 7, 10 |
|  | Limitations characterized? |  | Intervention stated? |  |
|  | Declaration of interest reported? |  | Comparator stated? |  |
|  |  |  | Outcome stated? |  |
|  |  |  | Limitations stated (general)? |  |
|  |  |  | Conflict of interest statement included? |  |
|  |  |  | Funding source stated? |  |
|  |  |  | Relevance to practice/policy and intended constituency/intended user stated? (optional) |  |
|  |  |  | Limitations on generalizability stated? (optional) |  |
| Comparator(s) | Comparator is standard of care? | 1, 2 | Comparator clearly stated? | 2, 4 |
|  | Non-intervention ("do-nothing" scenario) is an additional analysis? (optional) |  | Reported ICER? |  |
|  |  |  | Setting where comparator is administered stated? (optional) |  |
|  |  |  | Availability of comparator across population stated? (optional) |  |
| Evidence | Systematic review used? | 1, 2 | Parameter sources stated? | 2, 3 |
|  | Justification for single-study / trial estimates stated? (optional) |  | Parameter sources cited? |  |
|  |  |  | Areas where evidence lacks stated? (optional) |  |
| Measure of health outcome | DALYs as main outcome? | 1, 2 | Weighting methods stated? | 1, 2 |
|  | Alternate measures (QALYs, LYs, cases) reported as alternate analysis? (optional) |  | Outcomes not captured by DALYs stated? (optional) |  |
| Costs | Costs are true to reported perspective? | 2, 3 | Costs in local currency? | 2, 2 |
|  | Costs include implementation? |  | Costs in USD? |  |
|  | Out of pocket costs included? (optional) |  |  |  |
| Time horizon and discount rate | Lifetime time horizon used? | 3, 6 | Time horizon clearly stated? | 2, 4 |
|  | 3% discount rate used? |  | Discounting for both costs and outcomes clearly stated? |  |
|  | Discount rate used for costs and effects? |  | Justified use of shorter time horizon? (optional) |  |
|  | Shorter time horizon used as additional analysis? (optional) |  | Different discount rate for base case justified? (optional) |  |
|  | Different discount rate used as additional analysis to address government borrowings? (optional) |  |  |  |
|  | Different discount rate used as additional analysis to address time horizon greater than 30 years? (optional) |  |  |  |
| Perspective | Limited societal perspective used? | 2, 2 | Perspective clearly stated? | 1, 1 |
|  | Direct health costs reported? |  |  |  |
| Heterogeneity | Subgroup analysis performed/stated? | 1, 2 | Subgroup analysis performed/stated? | 1, 4 |
|  | Subgroup's difference in cost stated? (optional) |  | Choice of subgroup justified with evidence? (optional) |  |
|  |  |  | Subgroup's difference in effect stated? (optional) |  |
|  |  |  | Subgroups excluded due to lack of evidence stated? (optional) |  |
| Uncertainty | Structural sensitivity analysis performed? | 3, 3 | Reported results of sensitivity analysis? | 1, 3 |
|  | Sensitivity analysis of parameter source performed (deterministic)? |  | Likelihood of making wrong decision given evidence reported? (optional) |  |
|  | Sensitivity analysis of parameter precision performed (probabilistic)? |  |  |  |
| Budget impact | Budget impact assessment performed? | 1, 3 | Impact on budget stated? | 1, 5 |
|  | Budget impact reflects decision problem? (optional) |  | Budget impact includes implications for government and social insurance budgets? (optional) |  |
|  | Budget impact reflects constituency? (optional) |  | Budget impact includes implications for households and out of pocket expenses? (optional) |  |
|  |  |  | Budget impact includes implications for third-party payers? (optional) |  |
|  |  |  | Budget impact includes implications for external donors? (optional) |  |
| Equity considerations | Equity addressed at all in the paper? | 1, 2 | Influence of equity considerations stated in the paper? | 1, 2 |
|  | Equity assessed at all intervention stages? (optional) |  | Disproportionate effects of intervention stated? (optional) |  |
| DALY: disability-adjusted life year; QALY: Quality-adjusted life year; LY: life year; USD: United States dollar; ICER: incremental cost-effectiveness ratio.  Evaluation questions scored as either 0 (item not satisfied) or 1 (item satisfied), and are each weighted equally. Optional requirements noted with (optional) and are only included in sensitivity analysis scoring. | | | | |

**Table B: Reference case adherence scores by year** preventative interventions

|  |  | **Methodological  adherence score** | | | **Reporting  adherence score** | | |
| --- | --- | --- | --- | --- | --- | --- | --- |
| **Year** | **N** | **Mean (SD)** | **Min** | **Max** | **Mean (SD)** | **Min** | **Max** |
| 2011 | 44 | 61.8 (11.7) | 26.3 | 89.5 | 74.1 (8.4) | 47.6 | 90.5 |
| 2012 | 50 | 57.5 (9.9) | 31.6 | 84.2 | 71.7 (8.1) | 52.4 | 90.5 |
| 2013 | 44 | 57.7 (14.2) | 26.3 | 84.2 | 71.2 (10.8) | 42.9 | 90.5 |
| 2014 | 47 | 61.3 (10.4) | 31.6 | 84.2 | 73.7 (8.3) | 52.4 | 90.5 |
| 2015 | 78 | 60.7 (11.1) | 36.8 | 89.5 | 74.8 (8.8) | 47.6 | 90.5 |
| 2016 | 59 | 63.2 (10.5) | 31.6 | 78.9 | 76.1 (6.5) | 61.9 | 90.5 |
| 2017 | 76 | 56.0 (11.5) | 26.3 | 84.2 | 74.1 (7.9) | 57.1 | 90.5 |

**Table C: Reference case adherence raw scores by year, sponsor, and journal aspects**

|  |  | **Methodological  adherence raw score**  **(max 19)** | | | **Reporting  adherence raw score**  **(max 21)** | | |
| --- | --- | --- | --- | --- | --- | --- | --- |
|  | **N** | **Mean (SD)** | **Min** | **Max** | **Mean (SD)** | **Min** | **Max** |
| Base case analysis | 398 | 11.3 (2.2) | 5 | 17 | 15.5 (1.8) | 9 | 19 |
| Pre-post period^1^ |  |  |  |  |  |  |  |
| Pre-period: 2011-2013 | 138 | 11.2 (2.3) | 5 | 17 | 15.2 (1.9)* | 9 | 19 |
| Post-period: 2015-2017 | 213 | 11.3 (2.2) | 5 | 17 | 15.7 (1.7)* | 10 | 19 |
| Study sponsor^2^ |  |  |  |  |  |  |  |
| Academic | 53 | 11.3 (2.4) | 7 | 16 | 15.8 (1.7) | 11 | 19 |
| Government | 153 | 11.5 (2.1) | 6 | 17 | 15.8 (1.6) | 10 | 19 |
| Healthcare Org | 23 | 12.4 (2.1) | 6 | 17 | 16.2 (1.7) | 12 | 19 |
| Industry | 16 | 11.5 (2.1) | 6 | 14 | 15.7 (1.5) | 12 | 19 |
| Intergovernmental | 41 | 11.8 (2.0) | 7 | 16 | 15.6 (1.6) | 13 | 19 |
| Foundation | 56 | 11.4 (2.3) | 7 | 17 | 15.6 (1.7) | 12 | 19 |
| BMGF | 74 | 11.4 (2.1) | 6 | 16 | 15.8 (1.8) | 10 | 19 |
| Other | 24 | 11.1 (2.6) | 6 | 16 | 15.4 (1.9) | 11 | 19 |
| Cite reference case |  |  |  |  |  |  |  |
| Yes | 9 | 11.8 (2.4) | 9 | 17 | 16.6 (1.6) | 15 | 19 |
| No | 251 | 11.4 (2.1) | 5 | 16 | 15.7 (1.7) | 10 | 19 |
| Journal type |  |  |  |  |  |  |  |
| Clinical | 318 | 11.5 (2.1)* | 5 | 17 | 15.6 (1.7) | 10 | 19 |
| Non-clinical | 80 | 10.8 (2.5)* | 5 | 15 | 15.2 (2.1) | 9 | 19 |
| Journal impact factor^3^ |  |  |  |  |  |  |  |
| High | 336 | 11.5 (2.1)* | 5 | 17 | 15.6 (1.8) | 9 | 19 |
| Medium | 45 | 10.7 (2.4) | 5 | 14 | 15.3 (1.6) | 12 | 19 |
| Low | 12 | 9.5 (1.3)* | 8 | 12 | 14.8 (2.0) | 11 | 18 |
| *: Statistically significant difference (p<0.05) between categories (within methods/reporting requirements) per Student's t-test 1: Year 2014 was excluded from pre-post analysis to serve as dissemination period.  2: Categories are not mutually exclusive, t-test not calculated. 3: Journal impact factor categories defined by 2016 SCImago Journal Rank quartile: high = first quartile; medium = second quartile; low = third and fourth quartiles. Five journals' impact factors were not available. | | | | | | | |
